# Pharmacist-led new medicine service: a real-world cohort study in the Netherlands on drug-related problems, satisfaction, and self-efficacy in cardiovascular patients transitioning to primary care

**DOI:** 10.1007/s11096-024-01829-4

**Published:** 2024-12-10

**Authors:** Hendrik T. Ensing, Nelly Kurt, Ruby A. Janssen, Ellen S. Koster, Eibert R. Heerdink

**Affiliations:** 1 Outpatient Pharmacy ‘de Brug 24/7’, Zorggroep Almere, Almere, The Netherlands; 2 Department of Pharmacoepidemiology & Clinical Pharmacology, Utrecht Institute for Pharmaceutical Sciences (UIPS), Utrecht University, PO Box 80082, 3508 TB Utrecht, The Netherlands; 3 Institute of Paramedic Studies, HU University of Applied Sciences, Utrecht, The Netherlands; 4 Research Group Innovations in Pharmaceutical Care, HU University of Applied Sciences, Utrecht, The Netherlands; 5 Center of Education, Clinical Health Sciences, University Medical Center Utrecht, Utrecht, The Netherlands

**Keywords:** Community pharmacist, Drug-related problems, Medication counselling, Medication initiation, Primary care, Transitional care

## Abstract

**Background:**

Patients transitioning from hospital to home while starting long-term cardiovascular medicines are likely to experience drug-related problems (DRPs). The New Medicine Service (NMS) may support readmission to primary care.

**Aim:**

To evaluate NMS in a real world setting, for patients transitioning from hospital to primary care with newly prescribed cardiovascular medicines on identifying DRPs, patient satisfaction with medication information and patient self-efficacy. Secondary objectives were identifying risk factors for DRPs and assessing first-fill discontinuation.

**Method:**

A cohort study in an outpatient pharmacy and 14 community pharmacies in Almere, the Netherlands, involved patients ≥ 18 years receiving new cardiovascular prescriptions. Usual pharmacy care was complemented with a telephone counselling two weeks post-dispensing to identify and address DRPs. Patient satisfaction and self-efficacy were assessed during a follow-up call. First-fill discontinuation was measured using dispensing data, and logistic regression identified risk factors for DRPs.

**Results:**

Of 1647 eligible patients, 743 received NMS; 72.5% experienced ≥ 1 DRP. NMS improved patients’ satisfaction with information and self-efficacy (*p* < 0.001). Outpatient visits (adj. OR 0.64), cardiovascular medicine use (adj. OR 0.65), and use of chronic medicines (adj. OR 1.71) influenced DRPs. First-fill discontinuation remained unchanged post-NMS, but patients with DRPs discontinued more often (14.8% vs. 8.6%, *p* = 0.030).

**Conclusion:**

Implementing the NMS in a real-world transitional care setting allowed pharmacists to identify DRPs and provide counselling tailored to patient needs. Patients reported higher satisfaction with information and increased self-efficacy. Priority should be given to at-risk patients for DRPs, and deploy other pharmacy staff to perform the NMS.

**Supplementary Information:**

The online version contains supplementary material available at 10.1007/s11096-024-01829-4.

## Impact statements

- A pharmacist-led New Medicine Service was evaluated in a transitional care setting, demonstrating its feasibility in a real-world setting. The findings suggest a potential promising strategy for pharmacists to identify drug-related problems in patients initiating a cardiovascular medicine.
- Integrating the NMS in a transitional care setting improves patient satisfaction with the information they received regarding their new medicine and it can help reduce patients' self-efficacy challenges associated with integrating their new medicine in their everyday live.

## Introduction

Cardiovascular diseases are commonly managed with medications, but various barriers, including communication issues, can lead to poor adherence [1–3], especially in transitional care settings due to the involvement of multiple healthcare providers [4, 5]. Moreover, cardiovascular medicines typically have long-term prognostic goals often lacking immediate noticeable effects [6], leading to intentional or unintentional deviations from patients’ prescribed regimen [3, 7–9]. Studies show up to 19% of patients discontinue antihypertensive medication after the first fill, and up to 50% stop within the first six months [10–13].

Supporting patients’ adherence to medicines at the beginning of therapy is crucial. Addressing barriers is essential to optimise implementation over time. Adherence, in this context, refers to the extent to which a patient’s actual dosing matches the prescribed dosing regimen [14].

The New Medicine Service (NMS) is a pro-active, pharmacist-led intervention designed to support patients at therapy initiation, by identifying drug-related problems and providing tailored information and tools [15]. Studies on NMS have shown improvements in patient satisfaction, medication knowledge, and medication adherence, as well as cost-effectiveness [15–19].

However, only a few countries have integrated this service into usual pharmaceutical care provided [15, 16]. In the Netherlands, NMS has been studied in a randomized controlled trial in primary care, although results may not directly translate to real-world settings, let alone transitional care [17, 18]. Understanding barriers related to healthcare providers and patients is crucial, as they both play important roles in medication adherence [20]. Therefore, utilizing a living-lab approach to further study the NMS could enhance innovation development and implementation [21]. This approach involves real-world testing and iterative improvements based on stakeholder feedback.

### Aim

The primary objective of this study was to evaluate the NMS in a real-world setting, for patients transitioning from hospital to primary care with newly prescribed cardiovascular medicines on identifying DRPs, patient satisfaction with medication information and patient self-efficacy. Secondary objectives were identifying risk factors for DRPs and assessing first-fill discontinuation.

### Ethics approval

The study adhered to the Declaration of Helsinki principles and received approval from the Institutional Review Board of the Division of Pharmacoepidemiology and Clinical Pharmacology at Utrecht University (registration number UPF2009, date 28/09/2021).

Patient confidentiality and data protection were prioritized. All medical and pharmacy data were anonymized with unique patient IDs and stored on password-protected computers in the outpatient pharmacy. Access was restricted to two researchers (HE, NK) to maintain data security and integrity.

Informed consent was obtained from all individual participants included in the study. Patients were provided with information about the study’s purpose, procedures, potential risks and benefits, and their right to withdraw at any time without affecting their medical care.

This study was performed in line with the principles of the General Data Protection Regulation (GDPR) to ensure the protection of personal data.

## Method

### Study design and setting

A cohort study was conducted between April 2021 and August 2022, using a living lab approach [23]. Pharmacists, patients, and physicians collaborated to evaluate and adapt the NMS in everyday practice. The NMS was evaluated on several patient-reported outcome measures and first-fill discontinuation. Fidelity was promoted through an online self-report system, which tracked call duration, attempts, exclusions and was reviewed during monthly meetings [24]. In these meetings participants shared successes and helped others overcome barriers, leading to ongoing improvements, monitoring and facilitating the implementation progress.

The study was conducted at the outpatient pharmacy and 14 community pharmacies of Zorggroep Almere (ZGA), a large multidisciplinary primary healthcare organization in Almere, the Netherlands. ZGA’s community pharmacies are organized in three geographic clusters and gradually implemented the NMS. Simultaneously with the implementation of the NMS in the first cluster, patients in the two clusters not yet implementing NMS served as the control group, completing the *Satisfaction with Information about Medicines Scale (*SIMS) and *Medication Understanding and Use Self-Efficacy Scale (*MUSE, Supplementary material 5) [25, 26]. The outpatient pharmacy was based within and closely collaborates with the sole general non-teaching hospital (Flevoziekenhuis, 385 beds). City healthcare providers share a unified system for easy information exchange. Outpatient pharmacists have access to hospital files, enabling long-term patient monitoring.

### Participants

Community-dwelling patients ≥ 18 years who filled a new cardiovascular prescription intended for at least three months (defined as long term use), prescribed by a hospital physician at hospital discharge or an outpatient clinic were eligible for inclusion. Cardiovascular medication included: antithrombotic agents (ATC-code: B01), cardiac therapy (C01), diuretics (C03), beta blocking agents (C07), calcium channel blockers (C08), agents acting on the renin-angiotensin system (C09) and lipid modifying agents (C10). New cardiovascular prescription meant no dispensings for that medicine in the prior year. Patients using automated drug-dispensing systems or receiving pro re nata (PRN) prescriptions were excluded.

### New medicine service

**Fig. 1:**
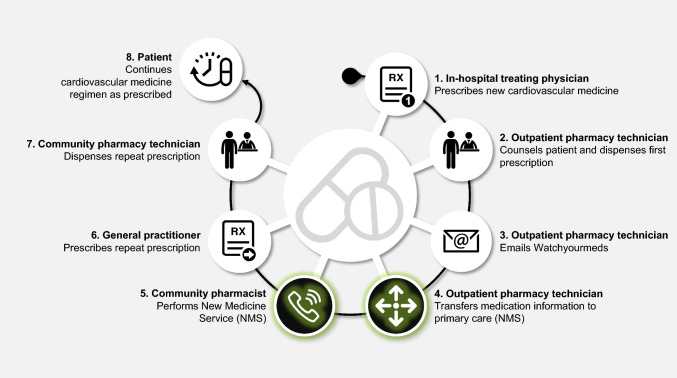
Usual care complemented with information transfer and the New Medicines Service (NMS)

NMS complemented usual pharmaceutical care (see Supplementary material 1 for a comprehensive description of both), consisting of (Fig. 1):

- *Information transfer* Patients’ medicine information was transferred to their GP and community pharmacist after visiting the outpatient pharmacy. The community pharmacist assessed patients’ eligibility for participation on a weekly basis using an automated generated list [27].
- *Telephone counselling* Eligible patients received a telephone call from their community pharmacist in the second week after starting their new cardiovascular medicine. A semi-structured interview protocol consisting of five open-ended questions with possible follow-up questions was used to identify practical and perceptual barriers to taking medicine (Supplementary material [28] 2).

### Definition of outcomes and data collection

Primary outcome measures were: (1) DRPs, (2) patients’ satisfaction with information received from the pharmacist and (3) patients’ medicine understanding and self-efficacy. Secondary outcomes were first-fill discontinuation and risk factors for DRPs.

### Drug-related problems

Community pharmacists collected data on DRPs using the TRIAGE protocol during telephone counselling [27]. The protocol included nine domains of DRPs: (1) practical intake problem, (2) problem with incorporating medicine in daily routine, (3) complexity of medicine regimen, (4) self-reported side effect, (5) failure to recognize necessity, (6) concern about medicine, (7) knowledge gap, (8) patient vulnerability and (9) costs. Pharmacists were also given follow-up actions to address identified DRPs, including providing advice, assessing discontinuation risk, consulting the prescriber, and providing written information or a medicine schedule [27].

### Satisfaction about medicines and self-efficacy

The day after telephone counselling, a pharmacy technician called the patient for follow-up questionnaires: SIMS and MUSE (Supplementary material 5) [25, 26]. The control group was contacted in the second week after starting their new cardiovascular medicine. SIMS (*action and usage* (scored 0–7) and *potential problems* (scored 0–8)) had a maximum score of 15, and MUSE (*taking medication* (scored 3–12) and *learning about medication* (scored 4–16)) had a maximum score of 28.

### Baseline characteristics and first-fill discontinuation

Patient characteristics and prescription data were obtained from pharmacy and hospital systems. A secondary outcome was the proportion of patients who discontinued their cardiovascular medicine after the first fill, defined as no refills within three months [12].

### Data coding and analysis

The target sample size was 200 NMS patients and 100 control patients. This size was chosen to reduce burden on pharmacy staff while ensuring statistical power to detect a clinically significant difference of 2 in the SIMS score at an alpha level of 0.05.

Baseline characteristics of patients in the NMS and control groups were compared using Mann–Whitney tests for numerical characteristics with non-normal distributions, and Chi-square tests for categorical variables. Logistic regression identified risk factors for DRPs. Akaike’s Information Criterion (AIC) and Hosmer and Lemeshow goodness-of-fit test were used to determine the best-fitting model. All data were analysed using SPSS statistics (v28.0.1.1), with *p* < 0.05 was considered significant.

## Results

### General characteristics

**Fig. 2:**
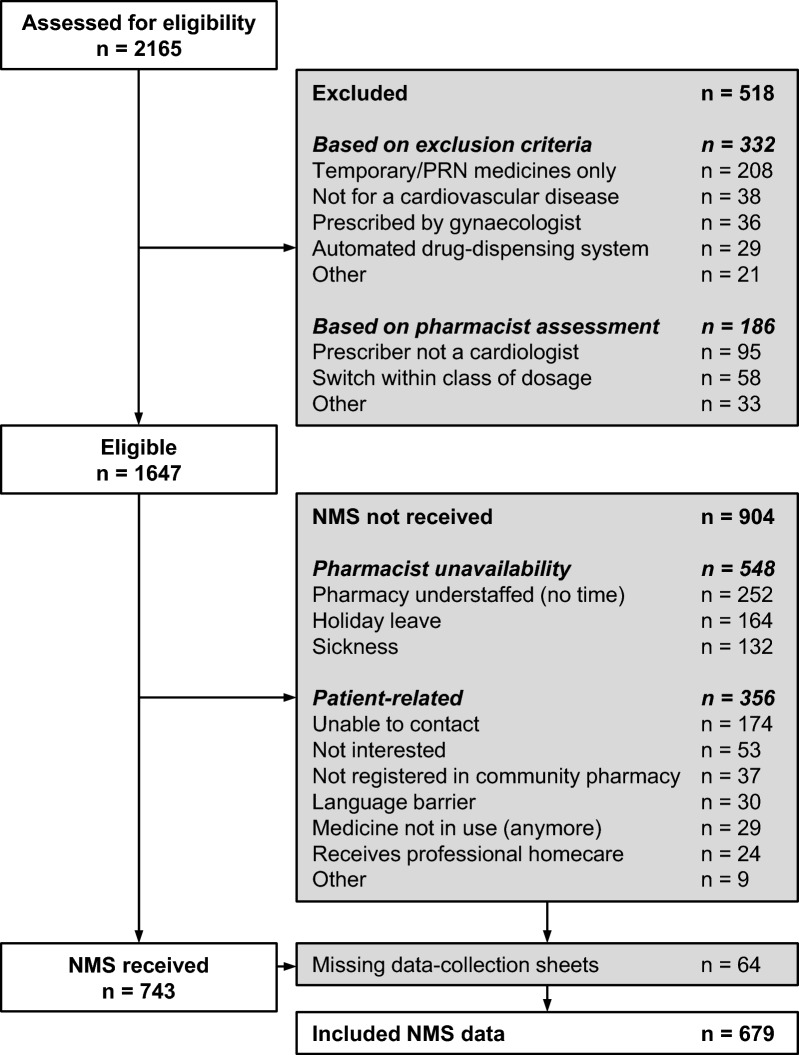
Patient flow chart for the NMS group

During the study, 2165 patients filled a first prescription for cardiovascular medicines, with 1647 (76.1%) being eligible for inclusion (Fig. 2). The main reasons for exclusion were temporary or *pro re nata* (PRN) prescriptions (9.6%, n = 208) and pharmacist assessment (8.6%, n = 186). Among the eligible, 743 (45.1%) patients received the NMS, averaging 12.3 ± 4.3 patients who received telephone counselling per week across the 14 pharmacies. In the NMS group, 240 questionnaires were completed (target was 200).

Of the eligible patients, 904 (54.9%) did not receive the NMS. Unavailability of the pharmacist (33.3%, n = 548) was the primary reason for patients not receiving counselling. Additionally, the NMS could not be provided to 356 patients (21.6%) due to patient-related factors, with the majority being unreachable by the pharmacist (10.6%, n = 174). In the control group, 96 questionnaires were completed out of the targeted 100 (Supplementary material 3).

Patient characteristics were similar between the NMS group and control group (Table 1). Patients were on average 63.6 ± 12.6 years old, with 44% (n = 327) female and 56% (n = 416) male. On average, the NMS was performed 13.5 ± 2.6 days (range 3–27) after first dispensing, lasting approximately 6 min (06:18 ± 03:30, range 1–24 min).

**Table 1:** Baseline sociodemographic, medical and medicine-related characteristics of the NMS and control group

Patient characteristic	NMS group (n = 743)	Control group (n = 96)	*P*-value
Female (%, (n))	44.0 (327)	47.9 (46)	0.469
Age, year (mean, (SD))	63.6 (12.6)	61.4 (13.5)	0.180
*Hospital department (%, (n))*			
Cardiology	54.5 (405)	37.5 (36)	0.109
Internal medicine	16.4 (122)	21.9 (21)	
Neurology	11.7 (87)	17.7 (17)	
Emergency	8.6 (64)	10.4 (10)	
Surgery	4.7 (35)	6.3 (6)	
Pulmonary	3.5 (26)	5.2 (5)	
Other	0.5 (4)	1.0 (1)	
*Cardiovascular (CV) medicines*			
Discharge prescription* (%, (n))	43.9 (326)	46.9 (45)	0.578
Outpatient clinic prescription (%, (n))	56.1 (417)	53.1 (51)	
CV medicines in use (mean, (SD, range))	1.9 (1.9, 0–9)	1.9 (1.8, 0–7)	0.850
CV medicines started (mean, (SD, range))	1.8 (1.1, 1–6)	1.7 (1.2, 1–6)	0.318
*Other prescription medicines in use*			
Other prescription medicines in use (mean, (SD, range))**	2.4 (2.4, 0–16)	2.4 (2.6, 0–17)	0.696

^*^ The other prescriptions originated from the outpatient clinics

^**^ Medicines for long-term use only, topical treatments excluded

Fewer patients (43.9%, n = 326) filled their prescription after hospital discharge compared to those who visited the outpatient clinic. Approximately two-thirds of the patients (67.0%, n = 498), had no cardiovascular medicines in use at time of dispensing the new prescription. Most patients (78.6%, n = 584) used other prescription medicines.

### Drug-related problems

A total of 1043 DRPs were identified for 679 patients (91.4% of all the patients who received the NMS (743), 64 were excluded due to missing data-collection sheets), with a mean number of 1.5 ± 1.5 DRPs per patient (range 0–9) (Table 2, Supplementary material 4). For 187 (27.5%) patients, pharmacists identified no DRPs, while 291 (42.9%) patients experienced multiple DRPs. The three major DRP categories were “Self-reported side effect” (28.7%, n = 299), “Complexity of medicine (regimen)” (25.7%, n = 268), and “Knowledge gap” (11.3%, n = 118) (Supplementary material 4). Patients discharged from the hospital (78.3%, n = 238 versus 67.7%, n = 254; p = 0.002) and those not previously using cardiovascular medicine (77.9%, n = 173 versus 69.8%, n = 319; p = 0.026) were more prone to experience a DRP.

**Table 2:** Number and categories of identified DRPs and performed follow-up actions to address that DRP by the pharmacist

DRP category	Identified DRPs (n = 1043) (%, (n))	Performed follow-up actions (n = 1711) (%, (n))
Practical intake problem	5.5 (57)	4.4 (76)
Problem with incorporating medicine in daily routine	4.6 (48)	3.2 (54)
Complexity of medicine (regimen)	25.7 (268)	16.6 (284)
Self-reported side effect	28.7 (299)	24.4 (418)
Fail to recognize the necessity of medicine	9.8 (102)	25.9 (443)
Concern about medicine	9.1 (95)	12.0 (206)
Knowledge gap	11.3 (118)	9.1 (156)
Vulnerable patient	5.1 (53)	4.1 (70)
Costs	0.3 (3)	0.2 (4)

The 1,043 DRPs resulted in 1711 follow-up actions by pharmacists, averaging 3.3 ± 2.1 per patient and 1.7 ± 1.0 per DRP. The most common issues were patients’ “Failure to recognize the necessity of their medicine” (25.9%, n = 443), “Self-reported side effect” (24.4%, n = 418), and “Complexity of medicine regimen” (16.6%, n = 284). Pharmacists provided counselling, reassurance, and guidance on medication management in response to these issues.

A multivariate regression analysis identified potential risk factors associated with DRPs (Table 3). Patients using prescription medicines for other comorbidities had an 1.71 times higher odds (95%-CI 1.10–2.66) of experiencing a DRP, compared to patients with no other medicines in use. Prescriptions from outpatient visits had an lower odds (OR 0.64, 95%-CI 0.43–0.94) of resulting in DRPs compared to prescriptions received after hospital discharge. Patients already using other cardiovascular medicines had lower odds (OR 0.65, 95%-CI 0.42–1.00) of experiencing a DRP. Finally, the odds of experiencing a DRP decreased with age (OR 0.98, 95%-CI 0.97–1.00 per year).

**Table 3:** Univariate and multivariable logistic regression analysis for patient characteristics and their effect on experiencing a DRP

Characteristic	Compared to	OR unadjusted (95% CI)	*P*-value unadjusted	OR adjusted (95% CI)	*P*-value adjusted
Age (years)	Age (years)	0.99 (0.97–1.00)	0.060	0.98 (0.97–1.00)	**0.025**
Female sex	Male sex	0.93 (0.66–1.30)	0.654	0.97 (0.69–1.38)	0.875
Outpatient clinic prescription	Hospital discharge prescription	0.58 (0.41–0.83)	**0.002**	0.64 (0.43–0.94)	**0.024**
Prescription from cardiology department	Prescription from other department	1.18 (0.84–1.66)	0.332	1.21 (0.84–1.74)	0.298
CV medicines in use	No CV medicines in use	0.66 (0.45–0.95)	**0.027**	0.65 (0.42–1.00)	**0.048**
> 1 CV medicines started	≤ 1 CV medicines started	1.40 (0.99–1.98)	0.054	1.11 (0.76–1.63)	0.298
Other prescription medicines in use	No other prescription medicines in use	1.52 (1.02–2.27)	**0.039**	1.71 (1.10–2.66)	**0.018**

### Satisfaction about medicine and self-efficacy

Pharmacy technicians collected 240 follow-up questionnaires for the NMS group and 96 for the control group (Table 4).

**Table 4:** SIMS and MUSE scores for the NMS and control group

Scores (median, [IQR])	NMS group (n = 240)	Control group (n = 96)	*P*-value
SIMS score	13 [10–15]	10 [7–12]	** < 0.001**
Action and Usage	7 [6, 7]	5.5 [4–6.75]	** < 0.001**
Potential problems	7 [4–8]	4.5 [2–6.75]	** < 0.001**
MUSE score	22 [21–28]	21 [20, 21]	** < 0.001**
Taking medication	9 [9–12]	9 [9–9]	**< 0.001**
Learning about medication	12 [12–16]	12 [12–12]	**< 0.001**

Overall, patients who received the NMS were more satisfied with the information they received, with a median score of 13 (IQR 10–15) versus 10 (IQR 7–12) for the control group (*p* < 0.001). They also scored higher on both subscales: *action and usage* (7 (IQR 6–7) versus 5.5 (IQR 4–6.75)) and *potential problems* (7 (IQR 4–8) versus 4.5 (IQR 2–6.75)) (*p* < 0.001).

Patients who received the NMS reported higher medicine understanding and self-efficacy (median score of 22 (IQR 21–28) versus 21 (IQR 20–21) (*p* < 0.001)) including both scores for *taking medication* (9 (9–12) versus 9 (9–9)) and *learning about medication* (12 (IQR 12–16) versus 12 (IQR 12–12)) subscales (*p* < 0.001). Experiencing a DRP did not affect SIMS and MUSE scores.

### First-fill discontinuation

Patients in the NMS group and control group were comparable regarding first fill discontinuation (13.3% versus 12.5%, *p* = 0.822) and late collection of their first refill (3.0% versus 4.2%, *p* = 0.521). Patient who experienced a DRP were more likely to discontinue their medicine after their initial first fill compared to patients who did not experience a DRP (14.8% versus 8.6%, *p* = 0.030). There was no difference in late refills (3.9% versus 1.1%, *p* = 0.060). Finally, the SIMS and MUSE scores were comparable between patients who discontinued or continued after their first fill (*p* = 0.293 and *p* = 0.373, respectively).

## Discussion

### Statement of key findings

This study demonstrated that implementing the NMS in a real-world setting where patients transitioned from hospital to primary care enabled pharmacists to identify DRPs in the majority of patients and provide additional counselling. Furthermore, patients were more satisfied with the information received about their medicine and reported higher self-efficacy and medicine understanding. Those at higher risk of DRPs were recently discharged from the hospital, new to cardiovascular medication, taking other prescriptions, and younger. However, the NMS did not improve first-fill discontinuation.

Previous studies align with the findings of this study, showing that DRP frequency varies by classification, patient population, medication type, and follow-up time [29]. Primary care studies show that many patients, starting new cardiovascular medicines, experience DRPs, including side effects, practical problems, and information needs [5, 9, 19].

At care transitions, adequate patient communication is crucial to ensure continuity of care and reduce DRPs [30]. Patient-provider communication is known to be an important predictor of patient satisfaction [31, 32]. Providing adequate pharmacy counselling and detailed medication information can improve patients’ knowledge, beliefs, adherence [33, 34], and attitudes towards services aimed at improving medication outcomes [19, 34].

Patients only learn how a medicine affects them once they start taking it, possibly leading to new questions and concerns about necessity, side effects, and long-term consequences. These issues influence adherence, but cannot be addressed in standard pharmaceutical care at first dispensing [2]. Integrating the NMS in patients’ healthcare journey before the second dispensing might therefore reduce first-fill discontinuation. This study showed that the NMS, when added to usual care, effectively fills that information gap, improving patients’ self-efficacy and understanding of their medicine, similar to other telephone-based strategies [35]. Although it is a relatively short intervention, possibly limiting a sustained effect, it can serve as a valuable starting point for the pharmacist to track patients’ drug-related problems. Moreover, telephone follow-up is a flexible, cost-effective way to improve patient adherence [36]. However, about a quarter of patients did not receive the NMS due to pharmacist time constraints (15.3%) or assessments (8.6%). Different beliefs about service goals among pharmacists can affect fidelity to NMS [37]. Several efforts were made to facilitate implementation, including engagement with the pharmacists, co-creation and piloting of the protocol, and monthly monitoring meetings to discuss and solve implementation barriers [37, 38]. However, these efforts alone seemed insufficient. Therefore, deploying well-trained pharmacy practitioners or pharmacy technicians to perform the NMS together with pharmacists could be a potential facilitator to overcome time constraints and increase patient counselling [17].

To further increase efficiency, pharmacists should identify patients most likely to benefit from the NMS by considering their risk factors for DRPs. This study found that patients experiencing a DRP were more likely to discontinue medication after the first fill [39]. Consistent with other studies, patients using non-cardiovascular medicines, indicating regimen complexity and comorbidity, were at higher risk for DRPs [12, 13]. Additionally, younger patients and those discharged from the hospital who had not used cardiovascular medicines before were more at risk. The counterintuitive result for younger patients (Table 3), albeit small, might be linked to the substantial number of problems identified in the complexity of medication regimen domain (Table 2). These could present challenges to (younger) patients with a more active lifestyle.

### Strengths and limitations

This study’s main strength was its real world setting, providing insights into the practical implementation of the NMS. Potential selection bias due to pharmacists’ professional assessments was minimized by providing a limited-information selection list and monthly reviews of exclusion reasons. Additionally, patients with positive attitudes towards pharmaceutical care may have been more likely to participate in follow-up, potentially overestimating satisfaction and self-efficacy, though this likely affected both groups equally, and a significant effect was still observed.

The study aimed to evaluate the NMS’s real-world impact without standardizing intervention delivery. Pharmacists shared their experiences and barriers during scheduled meetings. Lastly, pharmacy technician contact with the control group might have increased awareness of medication effects, potentially influencing adherence and reducing differences between groups.

### Interpretation

This study is the first to implement the NMS in transitional care using a pragmatic living-lab approach [21]. By involving pharmacists in the design and real-life testing of the intervention, the study addressed challenges and enhanced generalizability for real-world practices.

The evaluation aimed to assess the NMS’s effectiveness and feasibility in supporting patients during medication initiation after hospital discharge or outpatient visits, using real-world data to inform clinical practice and policy decisions on pharmacist-led interventions in transitional care.

### Further research

Earlier studies show the applicability of the NMS to other medicines as well; therefore, the NMS performed in this setting could serve as the basis for an extended follow-up adherence intervention. Additionally, a cost-effectiveness analysis could enhance the evaluation of the intervention [17, 40].

## Conclusion

Implementing the NMS in a real-world transitional care setting enabled pharmacists to identify DRPs and provide tailored counselling. Patients were more satisfied with the information and had higher self-efficacy after counselling. This shows that the NMS is valuable for patients starting cardiovascular medicines at readmission to primary care. Pharmacist unavailability was the main reason for patients not receiving the NMS. Pre-selecting high-risk patients and involving other pharmacy staff to perform the NMS might be warranted to improve implementation and reach more patients who could benefit from this service.

## Supplementary Information

Below is the link to the electronic supplementary material.

Supplementary file1 (DOC 34 KB)

Supplementary file2 (DOC 29 KB)

Supplementary file3 (PPT 136 KB)

Supplementary file4 (DOC 60 KB)

Supplementary file5 (DOC 49 KB)

## References

[1] Shoemaker SJ, de Oliveira DR. Understanding the meaning of medications for patients: the medication experience. Pharm World Sci. 2007;30:86–91. 17653833 10.1007/s11096-007-9148-5 PMC2082655

[2] Horne R, Chapman SC, Parham R, et al. Understanding patients’ adherence-related beliefs about medicines prescribed for long-term conditions: a meta-analytic review of the Necessity-Concerns Framework. PLoS ONE. 2013;8(12): e80633. 10.1371/journal.pone.0080633. 24312488 10.1371/journal.pone.0080633 PMC3846635

[3] van Geffen ECG, Philbert D, van Boheemen C, et al. Patients’ satisfaction with information and experiences with counseling on cardiovascular medication received at the pharmacy. Patient Educ Couns. 2011;83:303–9. 21550196 10.1016/j.pec.2011.04.004

[4] Ensing HT, Stuijt CC, van den Bemt BJ, et al. Identifying the optimal role for pharmacists in care transitions: a systematic review. J Manag Care Spec Pharm. 2015;21:614–36. 26233535 10.18553/jmcp.2015.21.8.614 PMC10397897

[5] Barber N, Parsons J, Clifford S, et al. Patients’ problems with new medication for chronic conditions. Qual Saf Health Care. 2004;13:172–5. 15175485 10.1136/qshc.2003.005926 PMC1743839

[6] Levine GN, Bates ER, Blankenship JC, et al. ACC/AHA/SCAI focused update on primary percutaneous coronary intervention for patients with ST-elevation myocardial infarction: an update of the 2011 ACCF/AHA/SCAI guideline for percutaneous coronary intervention and the 2013 ACCF/AHA guideline for the management of ST-elevation myocardial infarction: a report of the American College of Cardiology/American Heart Association task force on clinical practice guidelines and the society for cardiovascular angiography and interventions catheter. Cardiovasc Interv. 2015;87:1001–19. 10.1002/ccd.26325 26489034

[7] Burkhart PV, Sabaté E. Adherence to long-term therapies. Evidence for action. J Nurs Scholarsh. 2003;35:207. 14562485

[8] Linn AJ, Van Weert JC, Schouten BC, et al. Words that make pills easier to swallow: a communication typology to address practical and perceptual barriers to medication intake behavior. Patient Prefer Adherence. 2012;6:871–85. 23271896 10.2147/PPA.S36195 PMC3526884

[9] Ensing HT, Schulte RA, Koster ES, et al. Implementing a newly prescribed cardiovascular medicine in daily routine: the patient perspective at readmission to primary care. Res Soc Adm Pharm. 2023;19:293–300. 10.1016/j.sapharm.2022.10.003 36266176

[10] Ozaki AF, Choi AS, Le QT, et al. Real-world adherence and persistence to direct oral anticoagulants in patients with atrial fibrillation. Circ Cardiovasc Qual Outcomes. 2020;13: e005969. 32148102 10.1161/CIRCOUTCOMES.119.005969

[11] Leslie KH, McCowan C, Pell JP, et al. Adherence to cardiovascular medication: a review of systematic reviews. J Public Health (Oxf). 2019;41(1):e84-94. 29850883 10.1093/pubmed/fdy088 PMC6459362

[12] Evans CD, Eurich DT, Remillard AJ, et al. First-fill medication discontinuations and nonadherence to antihypertensive therapy: an observational study. Am J Hypertens. 2012;25:195–203. 22089108 10.1038/ajh.2011.198

[13] Lemstra M, Blackburn D. Nonadherence to statin therapy: discontinuation after a single fill. Can J Cardiol. 2012;28:567–73. 22658124 10.1016/j.cjca.2012.03.018

[14] Vrijens B, De Geest S, Hughes DA, et al. A new taxonomy for describing and defining adherence to medications. Br J Clin Pharmacol. 2012;73:691–705. 22486599 10.1111/j.1365-2125.2012.04167.x PMC3403197

[15] Elliot RA, Boyd MJ, Waring J, et al. Understanding and Appraising the New Medicines Service in the NHS in England (029/0124). Nottingham University School of Pharmacy, Department of Health Policy Research Programme Project. (PDF) Understanding and Appraising the New Medicines Service in the NHS in England: A randomised controlled trial and economic evaluation with qualitative appraisal comparing the effectiveness and cost effectiveness of the New Medicine Service in community pharmacies in England. https://www.fundingawards.nihr.ac.uk/award/029/0124 Accessed 18 Oct 2024.

[16] Kaae S, Dam P, Rossing C, et al. Evaluation of a pharmacy service helping patients to get a good start in taking their new medications for chronic diseases. Res Soc Adm Pharm. 2016;12:486–95. 10.1016/j.sapharm.2015.08.002 26319619

[17] Kooij MJ, Heerdink ER, van Dijk L, et al. Effects of telephone counseling intervention by pharmacists (TelCIP) on medication adherence; results of a cluster randomized trial. Front Pharmacol. 2016;7:269. 27625605 10.3389/fphar.2016.00269 PMC5003869

[18] Kooij MJ, Van Geffen ECG, Heerdink ER, et al. Patients’ general satisfaction with telephone counseling by pharmacists and effects on satisfaction with information and beliefs about medicines: results from a cluster Effects of telephone counseling intervention by pharmacists (TelCIP) on medication adherence; randomized trial. Patient Educ Couns. 2015;98:797–804. 25791373 10.1016/j.pec.2015.02.020

[19] Bremer S, Henjum S, Saether EM, et al. Drug-related problems and satisfaction among patients receiving pharmacist-led consultations at the initiation of cardiovascular drugs. Res Soc Adm Pharm. 2022;18:3939–47. 10.1016/j.sapharm.2022.06.005 35750567

[20] Astbury JL, Gallagher CT. Moral distress among community pharmacists: causes and achievable remedies. Res Soc Adm Pharm. 2020;16:321–8. 10.1016/j.sapharm.2019.05.019 31171433

[21] Zipfel N, Horreh B, Hulshof CTJ, et al. The relationship between the living lab approach and successful implementation of healthcare innovations: an integrative review. BMJ Open. 2022;12: e058630. 35768105 10.1136/bmjopen-2021-058630 PMC9240880

[22] von Elm E, Altman DG, Egger M, et al. The Strengthening the Reporting of Observational Studies in Epidemiology (STROBE)statement: guidelines for reporting observational studies. J Clin Epidemiol. 2008;61:344–9. 18313558 10.1016/j.jclinepi.2007.11.008

[23] Hogervorst S, Vervloet M, Janssen RA, et al. Implementing medication adherence interventions in four Dutch living labs; context matters. BMC Health Serv Res. 2023;23:1030. 37752529 10.1186/s12913-023-10018-4 PMC10523767

[24] Borrelli B. The assessment, monitoring, and enhancement of treatment fidelity in public health clinical trials. J Public Health Dent. 2011;71(Suppl 1):S52-63. 21656954

[25] Horne R, Hankins M, Jenkins R, et al. The Satisfaction with Information about Medicines Scale (SIMS): a new measurement tool for audit and research. Qual Health Care. 2001;10:135–40. 11533420 10.1136/qhc.0100135.. PMC1743429

[26] Cameron KA, Ross EL, Clayman ML, et al. Measuring patients’ self-efficacy in understanding and using prescription medication. Pat Educ Couns. 2010;80:372–6. 10.1016/j.pec.2010.06.029 PMC3184839 20650594

[27] Vervloet M, Zwikker HE, Linn AJ, et al. The development and proof of principle test of TRIAGE: a practical question set to identify and discuss medication-related problems in community pharmacy. Pharmacy (Basel). 2020;8:178. 32992492 10.3390/pharmacy8040178 PMC7711982

[28] Kooij MJ, Van Geffen ECG, Heerdink ER, et al. Effects of a TELephone Counselling Intervention by Pharmacist (TelCIP) on medication adherence, patient beliefs and satisfaction with information for patients starting treatment: study protocol for a cluster randomized controlled trial. BMC Health Serv Res. 2014;14:219. 24885317 10.1186/1472-6963-14-219 PMC4050986

[29] Krahenbühl-Melcher A, Schlienger R, Lampert M, et al. Drug-related problems in hospitals: a review of the recent literature. Drug Saf. 2007;30:379–407. 17472418 10.2165/00002018-200730050-00003

[30] Coleman EA, Berenson RA. Lost in transition: challenges and opportunities for improving the quality of transitional care. Ann Intern Med. 2004;141:533–6. 15466770 10.7326/0003-4819-141-7-200410050-00009

[31] Mast MS. On the importance of nonverbal communication in the physician-patient interaction. Patient Educ Couns. 2007;67:315–8. 17478072 10.1016/j.pec.2007.03.005

[32] Zhang H, Wang W, Haggerty J, et al. Predictors of patient satisfaction and outpatient health services in China: evidence from the WHO SAGE survey. Fam Pract. 2020;37:465–72. 32064515 10.1093/fampra/cmaa011 PMC7474531

[33] Sheils E, Tillett W, James D, et al. Changing medication-related beliefs: a systematic review and meta-analysis of randomized controlled trials. Health Psychol. 2023;43:155 10.1037/hea0001316. 37870789 10.1037/hea0001316

[34] Schackmann L, Koster ES, van Dijk L, et al. Communication skills-based training about medication switch encounters: pharmacy staff and patients’ experiences. Int J Clin Pharm. 2024;46:439–50. 38231348 10.1007/s11096-023-01664-z

[35] Chen C, Li X, Sun L, et al. Post-discharge short message service improves short-term clinical outcome and self-care behaviour in chronic heart failure. ESC Heart Fail. 2019;6:164–73. 30478888 10.1002/ehf2.12380 PMC6352960

[36] Elliott RA, Tanajewski L, Gkountouras G, et al. Cost effectiveness of support for people starting a new medication for a long-term condition through community pharmacies: an economic evaluation of the New Medicine Service (NMS) compared with normal practice. Pharmacoeconomics. 2017;35:1237–55. 28776320 10.1007/s40273-017-0554-9 PMC5684280

[37] Latif A, Waring J, Watmough D, et al. Examination of England’s New Medicine Service (NMS) of complex health care interventions in community pharmacy. Res Soc Adm Pharm. 2016;12:966–89. 10.1016/j.sapharm.2015.12.007 26806858

[38] Ensing HT, Koster ES, Sontoredjo TAA, et al. Pharmacists’ barriers and facilitators on implementing a post-discharge home visit. Res Soc Adm Pharm. 2017;13:811–9. 10.1016/j.sapharm.2016.08.003 27663391

[39] Karter AJ, Parker MM, Moffet HH, et al. New prescription medication gaps: a comprehensive measure of adherence to new prescriptions. Health Serv Res. 2009;44:1640–61. 19500161 10.1111/j.1475-6773.2009.00989.x PMC2754552

[40] Boyd M, Waring J, Barber N, et al. Protocol for the New Medicine Service Study: a randomized controlled trial and economic evaluation with qualitative appraisal comparing the effectiveness and cost effectiveness of the New Medicine Service in community pharmacies in England. Trials. 2013;1(14):411. 10.1186/1745-6215-14-411 PMC4220816 24289059

