# Supplementary material for: Pharmacist-led new medicine service: a real-world cohort study in the Netherlands on drug-related problems, satisfaction, and self-efficacy in cardiovascular patients transitioning to primary care
Source: Int J Clin Pharm. 2024 Dec 10;47(2):325–34. doi: 10.1007/s11096-024-01829-4 (PMC11920310; doi:10.1007/s11096-024-01829-4)
Supplement: Supplementary file 1 — Supplementary file1 (DOC 34 KB) [file 11096_2024_1829_MOESM1_ESM.doc]

**Supplementary material 1.** Dutch usual healthcare and the adoption of the New Medicine Service (NMS) in this setting

Usual care at Dutch outpatient and community pharmacies

***Clinical risk management***

In the Netherlands, clinical risk management is an integral part of pharmacies aimed at ensuring patient safety and the effective management of medication therapy. It includes several key components, such as: **electronic prescribing** which reduces the risk of transcription errors and ensures that prescriptions are clear and legible, **medication reviews** which helps to identify potential drug interactions, dosing errors, and other risks, and **patient counselling**. All Dutch pharmacies keep electronic dispensing data and professional guidelines emphasize the importance of counselling at the start of a new medicine, the latter is mainly performed by a pharmacy technician. Furthermore, Dutch pharmacies work closely with other healthcare providers, including general practitioners (GPs) and hospitals, to ensure a coordinated approach to patient care.

***Patients’ healthcare journey***

In this study, usual care started with a patient filling a first prescription at the outpatient pharmacy (Figure 1). This new medicine was prescribed by an in-hospital treating physician either at hospital discharge or at the outpatient clinic. In addition to the Patient Information Leaflets (PILs) of the medication, the outpatient pharmacy sent an animated and spoken medicine information video to the patient by email (Watchyourmeds, Figure 1) [24]. Usual care continued when patients requested a repeat prescription from their general practitioner after approximately one month, the readmission to primary healthcare. The community pharmacy dispensed that prescription and patients continued their cardiovascular medicine in primary care as prescribed.

**Adoption of the New Medicine Service in Dutch living lab Almere**

The interview protocol, consisting of five open-ended questions with possible follow-up questions was used to identify practical and perceptual barriers to taking medicine (Supplementary material 2). The protocol was updated for this study in collaboration with the original researchers of the NMS in the Netherlands (Kooij et al) and comprised of two instruments: (1) *TRIAGE* practical questions set and trigger list for identification of possible drug-related problems (DRP) and the (2) professional Calgary-Cambridge guideline for pharmacy counselling (Royal Dutch Pharmacy Association, KNMP) [25,26]. Next, an expert panel, consisting of four local community pharmacists and a communication expert in the living lab co-creation, commented on the applicability of the updated protocol in daily practice of Almere and on incorporating an open-ended communication style. All participating community pharmacists had experience performing patient counselling interviews and followed a one-day training course on patient-centred communication skills and types and causes of nonadherence provided by the same communication expert. Furthermore, to improve treatment fidelity and to ensure generalizability and robustness, pharmacists received protocol-specific training on applying the NMS protocol in everyday pharmacy practice by the research team and received a written manual. When a DRP was identified, the pharmacist provided information, counselling and reassurance, offered possible solutions or referred the patient to their prescriber.
