# Supplementary material for: Pharmacist-led new medicine service: a real-world cohort study in the Netherlands on drug-related problems, satisfaction, and self-efficacy in cardiovascular patients transitioning to primary care
Source: Int J Clin Pharm. 2024 Dec 10;47(2):325–34. doi: 10.1007/s11096-024-01829-4 (PMC11920310; doi:10.1007/s11096-024-01829-4)
Supplement: Supplementary file 2 — Supplementary file2 (DOC 29 KB) [file 11096_2024_1829_MOESM2_ESM.doc]

**Supplementary material 2.** Semi-structured interview protocol of the New Medicine Service.

**Initiation**

Aim: preparation, identify reason for consultation.

- *‘Hello, this is [name + position] from [pharmacy name]. Around [date] you received [name of medicine] from the outpatient pharmacy for the first time. As an additional* *pharmacy service**we call patient who have recently started a cardiovascular medicine to hear how they are doing. Is this a convenient time for you?’*

**Gathering information**

1. Aim: provide opportunity to **ask questions/express concerns**.

- Opening question: *‘Before I start, what questions or concerns do you have about your new medicine?’*

1. Aim: assess **intake problems**.

- Opening question: *‘Have you started taking your medicine yet? I am curious about your experiences, how have you been taking this medicine?’*

- Follow-up questions: ‘*Is there anything you find troublesome in using this medicine?’ ‘To what extent do you manage to take the medicine every day?’ How do you make sure you don’t forget to take your medicine?’ ‘What do you do in case you do forget to take your medicine?’*

1. Aim: assess **possible side effects**.

- Opening question: *‘Any medicine can also have side effects. What is that like for you? Which side effects do you think you experience?’*

- Follow-up questions: *‘Would that side effect be a reason for you to stop taking this medicine?’ We know this medicine can cause [side effect], have you also experienced this?*

1. Aim: assess **views** (need/concern) about the medicine and **motivation** for use.

- Opening question: *‘How do you feel about using this medicine (long-term)?’*

- Follow-up questions: ‘How important is it to you to use this medicine every day?’ ‘Which concerns do you have about this medicine?’ ‘What effect do you experience from the medicine?’ ‘What do you expect from this medicine?’ What would be a reason for you to stop taking this medicine?’’

1. Aim: provide final opportunity to assess **other questions/concerns.**

- Opening question: *‘Which questions or concerns do you still have for me?’*

**Explanation and planning**

Aim: provide counselling, accurate recall/understanding, shared decision making. Examples:

- *‘You indicated that you had a question/concern about …’/ ‘I will now address this’*
- *‘I am now going to explain [the effect] [the usage] [etc.] to you’*
- *‘I would like to know if I have explained it correctly. Can you tell me how you will take the medicine later?’ (feedback method)*

**Closing**

Aim: ensure point of closure, future planning, log counselling.

- *‘We discussed the following: …’*
- *‘If you have any questions about your medicine later, please contact me.’*
