# Supplementary material for: Pharmacist-led new medicine service: a real-world cohort study in the Netherlands on drug-related problems, satisfaction, and self-efficacy in cardiovascular patients transitioning to primary care
Source: Int J Clin Pharm. 2024 Dec 10;47(2):325–34. doi: 10.1007/s11096-024-01829-4 (PMC11920310; doi:10.1007/s11096-024-01829-4)
Supplement: Supplementary file 3 — Supplementary file3 (PPT 136 KB) [file 11096_2024_1829_MOESM3_ESM.ppt]

## Slide 1
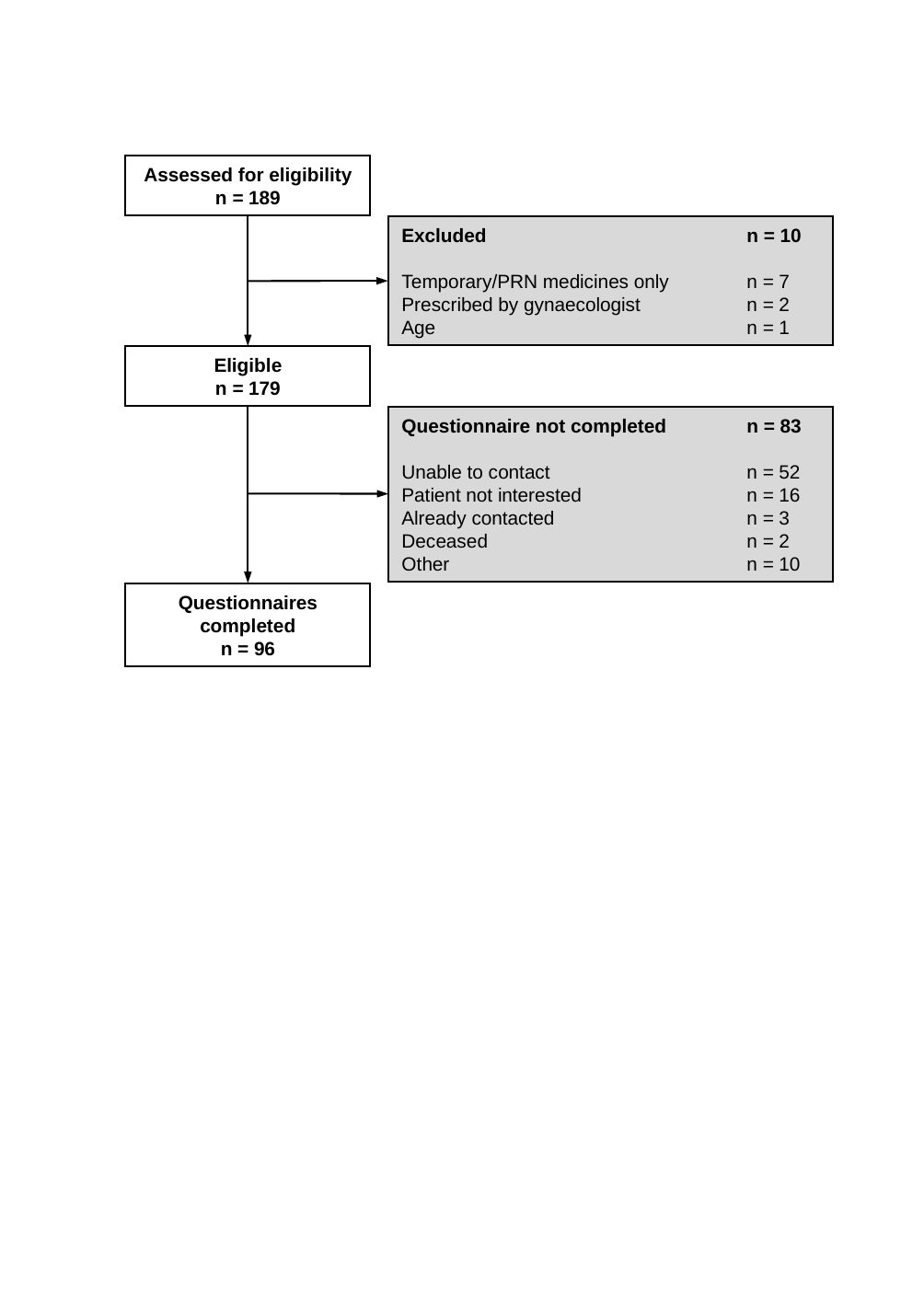

Assessed for eligibility
n = 189
Excluded 				n = 10
Temporary/PRN medicines only		n = 7
Prescribed by gynaecologist		n = 2
Age 					n = 1
Eligible
n = 179
Questionnaire not completed		n = 83
Unable to contact			n = 52
Patient not interested			n = 16
Already contacted			n = 3
Deceased 				n = 2
Other 					n = 10
Questionnaires completed
n = 96
