# Supplementary material for: Pharmacist-led new medicine service: a real-world cohort study in the Netherlands on drug-related problems, satisfaction, and self-efficacy in cardiovascular patients transitioning to primary care
Source: Int J Clin Pharm. 2024 Dec 10;47(2):325–34. doi: 10.1007/s11096-024-01829-4 (PMC11920310; doi:10.1007/s11096-024-01829-4)
Supplement: Supplementary file 4 — Supplementary file4 (DOC 60 KB) [file 11096_2024_1829_MOESM4_ESM.doc]

**Supplementary material 4**. Number and categories of identified DRPs (n = 1043, *left*) and performed follow-up actions (n = 1711, *right*) to address that DRP by the pharmacist (extended).

| **Practical intake problem (%,(n))** | | | |
| --- | --- | --- | --- |
| *Total identified DRPs* | *5.5% (57)* | *Total follow-up actions* | *4.4% (76)* |
| Unclear medicine regimen | 2.4% (25) | Recommend medicine distribution system | 1.7% (29) |
| Forgetfulness | 1.6% (17) | Provide intake schedule | 1.1% (19) |
| Other (e.g. problem preparing medicine) | 1.4% (15) | Other (e.g. advise tool, provide medicine reminder chart) | 1.6% (28) |
| **Problem with incorporating medicine in daily routine (%,(n))** | | | |
| *Total identified DRPs* | *4.6% (48)* | *Total follow-up actions* | *3.2% (54)* |
| Unable to keep stock | 3.7% (39) | Advise repeat prescription service | 2.4% (41) |
| Other (e.g. problem with routine interruptions) | 0.9% (9) | Other (e.g. provide written intake schedule) | 0.8% (13) |
| **Complexity of medicine (regimen) (%,(n))** | | | |
| *Total identified DRPs* | *25.7% (268)* | *Total follow-up actions* | *16.6% (284)* |
| Unclear repeat prescription process | 12.9% (135) | Explain repeat prescription process | 8.1% (138) |
| Unclear how long to take | 4.5% (47) | Counsel on duration/ changes | 3.5% (60) |
| Lost all track (Too much medicines) | 4.2% (44) | Other (e.g. advise medication distribution system) | 5.0% (86) |
| Other (e.g. complex regimen) | 4.0% (42) |  |  |
| **Self-reported side effect (%,(n))** | | | |
| *Total identified DRPs* | *28.7% (299)* | *Total follow-up actions* | *24.4% (418)* |
| Central nervous system | 11.7% (122) | Reassure patient | 11.7% (200) |
| Gastro-enteric system | 5.9% (62) | Assess risk for discontinuation | 9.2% (157) |
| Cardiovascular system | 3.0% (31) | Contact prescriber | 3.6% (61) |
| Other | 8.1% (84) |  |  |
| **Fail to recognize the necessity of medicine (%,(n))** | | | |
| *Total identified DRPs* | *9.8% (102)* | *Total follow-up actions* | *25.9% (443)* |
| Lack of noticeable effect | 5.4% (56) | Explain reason for medicine | 9.7% (166) |
| Other (e.g. questions the validity of diagnosis) | 4.4% (46) | Counsel on relevance of taking medicine as prescribed | 8.8% (151) |
|  |  | Other (e.g. counsel on not discontinuing independently) | 7.4% (126) |
| **Concerns about medicine (%,(n))** | | | |
| *Total identified DRPs* | *9.1% (95)* | *Total follow-up actions* | *12.0% (206)* |
| Concern about side effects | 6.6% (69) | Provide sympathetic ear | 5.9% (101) |
| Other (e.g. concern about long-term use or dependency) | 2.5% (26) | Provide reassurance on risk of side effect | 3.9% (66) |
|  |  | Other (e.g. assess if concern was adequately addressed) | 2.3% (39) |
| **Knowledge gap (%,(n))** | | | |
| *Total identified DRPs* | *11.3% (118)* | *Total follow-up actions* | *9.1% (156)* |
| Knowledge gap concerning disease or medicine | 8.1% (84) | Explain mechanism of action, side effects or how to use | 5.0% (86) |
| Received conflicting information from healthcare providers | 2.1% (22) | Emphasize relevance of using medicine regularly | 1.6% (28) |
| Other (e.g. lacks understanding of medicine regimen) | 1.2% (12) | Other (e.g. point the pharmacy website out to patient) | 2.5% (42) |
| **Vulnerable patient (%,(n))** | | | |
| *Total identified DRPs* | *5.1% (53)* | *Total follow-up actions* | *4.1% (70)* |
| Language barrier | 2.2% (23) | Assess and refer to patients’ support system | 1.9% (33) |
| Limited health literacy | 1.9% (20) | Use of conversation techniques | 2.2% (37) |
| Other | 1.0% (10) |  |  |
| **Costs (%,(n))** | | | |
| *Total identified DRPs* | *0.3% (3)* | *Total follow-up actions* | *0.2% (4)* |
