# Supplementary material for: Pharmacist-led new medicine service: a real-world cohort study in the Netherlands on drug-related problems, satisfaction, and self-efficacy in cardiovascular patients transitioning to primary care
Source: Int J Clin Pharm. 2024 Dec 10;47(2):325–34. doi: 10.1007/s11096-024-01829-4 (PMC11920310; doi:10.1007/s11096-024-01829-4)
Supplement: Supplementary file 5 — Supplementary file5 (DOC 49 KB) [file 11096_2024_1829_MOESM5_ESM.doc]

**Supplementary Material 5.** Medication Understanding and Use Self-Efficacy Scale (MUSE).

**MUSE questionnaire**

- Cameron KA, Ross EL, Clayman ML, Bergeron AR, Federman AD, Bailey SC, Davis TC, Wolf MS. Measuring patients' self-efficacy in understanding and using prescription medication. Patient Educ Couns. 2010 Sep;80(3):372-6.

|  |  | Taking/ learning | Strongly disagree | Disagree | Neither agree or disagree | Agree | Strongly agree |
| --- | --- | --- | --- | --- | --- | --- | --- |
| 1 | It is easy for me to take my medicine on time. | T |  |  |  |  |  |
| 2 | It is easy for me to ask my pharmacist questions about my medicine. | L |  |  |  |  |  |
| 3 | It is easy for me to understand my pharmacist’s instructions for my medicine. | L |  |  |  |  |  |
| 4 | It is easy for me to understand instructions on medicine bottles. | L |  |  |  |  |  |
| 5 | It is easy for me to get all the information I need about my medicine. | L |  |  |  |  |  |
| 6 | It is easy for me to remember to take all my medicines. | T |  |  |  |  |  |
| 7 | It is easy for me to set a schedule to take my medicines each day. | T |  |  |  |  |  |
| 8 | It is easy for me to take my medicines every day. | T |  |  |  |  |  |
